# Is it time for a new measurement approach? A closer look at the assessment of cognitive adaptability in complex problem solving

**DOI:** 10.3389/fpsyg.2015.01664

**Published:** 2015-10-28

**Authors:** Ronny Scherer

**Affiliations:** Faculty of Educational Sciences, Centre for Educational Measurement at the University of Oslo, University of OsloOslo, Norway

**Keywords:** adaptability, cognitive flexibility, complex problem solving, computer-based assessments, dynamic testing

## Introduction

In an ever-changing society, in which scientific knowledge increases rapidly, and workplace demands shift toward “twenty-first century skills,” the ability to adapt one's thinking, drive, and emotions to changing and novel problem situations has become essential (OECD, 2013). As a consequence, a number of researchers from different disciplines developed assessment tools that allow for inferences on the level of cognitive adaptability an individual is able to achieve (Martin and Rubin, 1995; Ployhart and Bliese, 2006; Birney et al., 2012; Martin et al., 2013; Colé et al., 2014). However, the limited evidence on construct validity that comes along with existing assessments that do not provide interactive and dynamic performance tasks points to the need of considering alternative assessment methods (Bohle Carbonell et al., 2014). With the advancement of computer-based assessments of complex problem solving (CPS) in educational contexts, new opportunities of measuring adaptability occur, which may overcome these challenges (Wood et al., 2009; Greiff and Martin, 2014). Specifically, the rich data obtained from such assessments, which, for instance, include information on response times, sequences of actions, and the navigation through the assessment), allow researchers to study cognitive adaptability in more depth, as they go beyond mere performance measures (i.e., correct vs. incorrect). The main message of this article consequently reads: It is time to exploit the potential of computer-based assessments of CPS in order to measure cognitive adaptability as a twenty-first century skill.

## Existing conceptualizations of cognitive adaptability

VandenBos (2007) defined adaptability as the “capacity to make appropriate responses to changed or changing situations; the ability to modify or adjust one's behavior in meeting different circumstances or different people” (p. 17). This capacity does not only involve cognitive and behavioral aspects but also affective adjustments to novelty and changes (Martin and Rubin, 1995; Martin et al., 2013). Existing research pointed out that the abilities to solve complex and ill-structured problems, to deal with uncertainty, and to adapt emotionally and culturally are essential facets of the construct (Pulakos et al., 2000). Nevertheless, cognitive psychology often refers adaptability to “cognitive flexibility,” describing the general ability to deal with novelty (Beckmann, 2014). When students face novelty or changes in a problem situation, a number of processes need to take place (Ployhart and Bliese, 2006): First, students have to recognize that there is novel information or changes. Second, they have to decide whether or not the novel information or the changes are relevant for the problem situation. Third, the scientific credibility and validity of the information must be evaluated. Finally, it needs to be decided whether or not their strategies to solve the problem need to be adjusted. Although, the problem situation may change drastically such that the number of variables and their connections change, adaptability does not necessarily require students to change their strategies. By contrast, novel information about the problem structure, the goals to achieve, or the context of the problem may require different strategies, particularly when students have to extract or generate information from sources other than the ones provided (Beckmann, 2014). In such situations, adaptability may be indicated by adjustments in strategic actions.

In light of the current conceptualizations of cognitive adaptability and the processes involved, it seems as if computer-based assessments have the potential to capture the many aspects of the construct (Gonzalez et al., 2013).

## Transferring approaches of measuring complex problem solving to adaptability

Because many real-life situations students face are complex and subject to novelty and change, studying adaptability in complex problem-solving situations provides a more realistic perspective on the construct than in simple problem situations (Jonassen, 2011). Moreover, many problems students are asked to solve in specific domains such as science comprise a number of variables that are connected in complex ways (Scherer, 2014). The ability to solve such complex problems by interacting with the problem environment, in which the information that is necessary in order to solve the problem is not given in the beginning of the problem solving process, refers to “complex problem solving” (CPS; Funke, 2010). In two recently published opinion papers, Funke (2014) and Greiff and Martin (2014) elaborated on the importance of CPS in educational and psychological contexts, and pointed out that the construct comprises two main dimensions (see also Greiff et al., 2013): First, students interact with the problem environment in order to acquire knowledge about the variables involved and their interconnections (knowledge acquisition). In this phase, a mental model about the problem situation is generated, which may be challenged by novel or changing information in the course of problem solving. As a consequence, students may revise and adapt their mental model. Second, the acquired knowledge is used to solve the problem, that is, to achieve a specific goal state (knowledge application).

**Figure 1:**
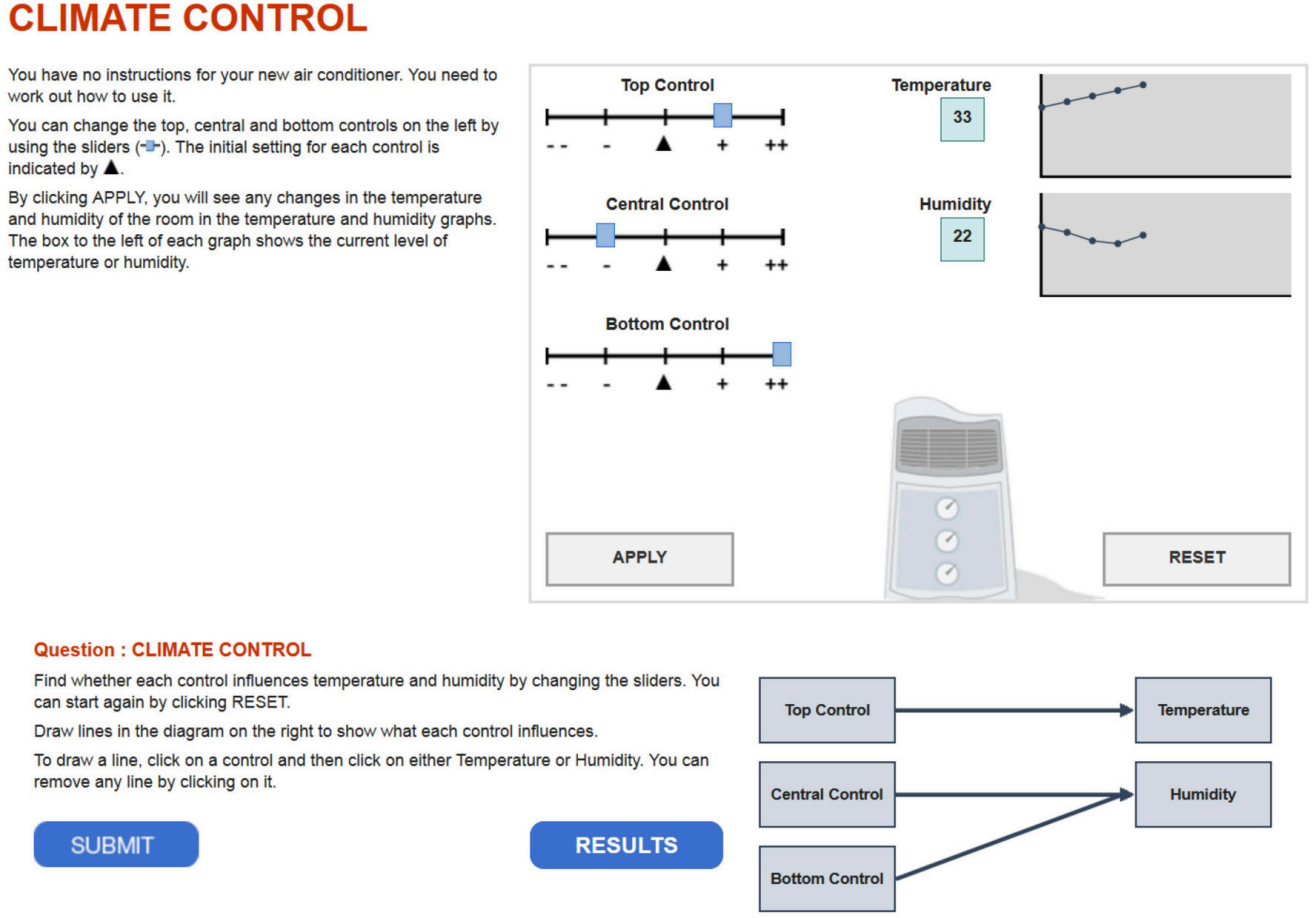
**Example of a CPS minimal complex system task administered in PISA 2012 (OECD, 2014)**. The figure has been retrieved from http://www.oecd.org/pisa/test/testquestions/question3/ [accessed 29/9/2015].

Although, CPS is difficult to measure, and many discussions emerged about the reliability and validity of CPS assessments (Danner et al., 2011; Scherer, 2015), Funke (2014) and Greiff and Martin (2014) pointed to two promising measurement approaches: *Computer-Simulated Microworlds (CSMs)* and *Minimal Complex Systems (MCSs)*. The former present students with a complex environment, that is comprised of a large number of variables such that the problem cannot be fully understood (Funke, 2010; Danner et al., 2011; Scherer et al., 2014). Given that CSMs allow for changes in the problem situation over time or as function of students' interactions with the problem, and thus present novel information to students, cognitive adaptability may be captured by these kinds of CPS assessments. This potential has promoted the concept of dynamic testing as a way to evaluate cognitive adaptability (Beckmann, 2014). Given that dynamic testing requires adjustments to novelty and changes, I argue that this feature has the potential to track adaptability in a straightforward way. For instance, situations may occur, which appear to be novel to the student; however, the acquired knowledge and skills enable a person to handle the situation appropriately. Other situations may occur, in which prior knowledge and skills are not sufficient and novel problem-solving approaches are needed. In such situations, adaptability can be evaluated by looking at students' reactions on dynamics and novelty. These reactions may manifest in *changes* of problem solving behavior or, if adjustments are not necessary, in the *stability* of problem solving behavior. For instance, changes in the number of variables in a problem situation may not require different problem solving strategies, because varying one variable at a time may still be a reasonable and goal-driving approach (Kuhn et al., 2008). In this respect, the advantages of computer-based assessments come into play. Specifically, it is generally possible to record students' actions in log-files, which contain not only information on the performance, but also on process data such as response times and sequences of actions. I believe that evaluating these rich data sources obtained from CSMs will make the concept of adaptability accessible to educational measurement. However, due to time-intense single tasks, the reliability and scalability of microworlds is severely threatened (Greiff et al., 2012; Scherer, 2015). As a response to this challenge, Greiff et al. (2013) developed MCSs to assess CPS with multiple independent tasks. Recent research has indicated their scalability in measuring CPS and its dimensions; and their enormous potential was recognized in the Programme for International Student Assessment (PISA) in 2012 (OECD, 2014). In MCSs, students are presented with a system (i.e., the problem environment) that simulates a specific scientific concept (e.g., climate control; Figure 1). Their first task is to generate knowledge about this system of variables and their relations by testing how changes in the input variables (e.g., top, central, and bottom control; Figure 1) affect the output variables (e.g., temperature, humidity; Figure 1). Students represent their mental model about these relations in a path diagram. Their second task is to apply this knowledge in a problem situation, where they have to reach a specific goal state or outcome value (e.g., specific levels of temperature and humidity). This task concludes their work on a minimal complex system and further MCSs may be administered subsequently. Given this design, MCSs allow for incorporating interactive, dynamic, and uncertain elements into the problem environment, but still provide sufficient psychometric characteristics in terms of reliability and validity (Greiff et al., 2013). As a consequence, the MCS approach qualifies for assessing students' cognitive adaptability.

Surprisingly, a systematic investigation of adaptability has not yet been conducted with these promising assessments, although it would be straightforward to transfer elements of dynamics and novelty from CSMs to MCSs in order to evaluate the students' reactions and adjustments. For instance, MCSs were used to assess students' complex problem-solving skills in PISA 2012. Students had to work on a sequence of MCSs, which differed in their characteristics such as the number of input and output variables, the relations among these variables, and the situational contexts (e.g., climate control vs. ticket machine; OECD, 2014). But given that too many of these characteristics in two adjacent MCSs were varied at the same time, a systematic investigation of adaptability may be compromised. In fact, adaptability is best examined when changes and novelty are systematically controlled in the test design. Moreover, the current MCSs in PISA 2012 do not contain a dynamic component, which manifests, for instance, in system changes over time without any interaction with the problem solver (so-called “eigendynamics”; Funke, 2010). Nevertheless, MCSs generally have the potential to assess cognitive adaptability, if these design elements are incorporated. In this regard, I can think of a number of scenarios: For instance, *after* the completion of a complex problem solving task, a new task can be presented which appears to be identical to the previous task; however, the connections between the variables, the number of variables, and whether or not they change dynamically over time have changed. In such a scenario, students would have to recognize the changes and adapt their problem solving behavior. In another possible scenario, novel information about the problem structure or the problem goal is presented *during* the problem solving process (Goode and Beckmann, 2010). Since this information may originate from different sources, students have to evaluate the information according to its credibility and relevance for the problem in addition to recognizing the new situation. All of these possible scenarios demand computer-based assessments that (1) are highly interactive tools to track not only students' cognitive performance but also their specific behavior; (2) contain a variety of data available to infer on adaptability (e.g., time, actions, performance; Pool, 2013); (3) allow for the incorporation of novelty and dynamics; (4) evaluate adaptability as change or stability across complex problem solving situations. In my opinion, these features will address the current need for valid assessments of adaptability, which marry the advantages of different, computer-based assessments of CPS (Beckmann, 2014).

## Conclusion

On the basis of the conceptualizations of adaptability and existing CPS assessments, the following conclusions is drawn: Marrying the two CPS assessments traditions, namely computer-simulated microworlds and minimal complex systems, by transferring elements of dynamic changes and novelty from CSMs to MCSs provides a potential approach to evaluate cognitive adaptability. These different aspects may guide researchers through the processes of developing valid assessments of the construct. There is the hope that, although adaptability is by no means considered to be a novel construct, the current innovations in computer-based assessments of CPS provide new ways to evaluate this essential twenty-first century skill.

### Conflict of interest statement

The author declares that the research was conducted in the absence of any commercial or financial relationships that could be construed as a potential conflict of interest.
